# Self-Powered Sensors for Environmental Monitoring

**DOI:** 10.3390/nano16090526

**Published:** 2026-04-27

**Authors:** Xiali Yang, Min Dai, Man Zhang, Shunyi Chen, Peng Zhang, Hancong Liu, Qitao Zhou, Jing Pan

**Affiliations:** State Key Laboratory of Geomicrobiology and Environmental Changes, Engineering Research Center of Nano-Geomaterials of Ministry of Education, Faculty of Materials Science and Chemistry, School of Mechanical Engineering and Electronic Information, China University of Geosciences, Wuhan 430074, China; m18634228327@163.com (X.Y.); 17872225431@163.com (M.D.); zm20010708@163.com (M.Z.); a1093734887@163.com (S.C.); 13452224788@163.com (P.Z.); m15155469755@163.com (H.L.); zhouqitao@cug.edu.cn (Q.Z.)

**Keywords:** triboelectric nanogenerator, self-powered, environmental monitoring, biochemical sensing

## Abstract

The development of self-powered environmental sensors is of great practical significance for addressing the power supply dilemma of traditional sensors in remote areas and avoiding environmental pollution from waste batteries. Given that the majority of the self-powered environmental sensors are based on the TENG principle, especially the active self-powered sensors, this paper reviews recent advances in triboelectric nanogenerator (TENG)-based self-powered environmental sensors. What distinguishes this review from the previous ones published on TENG is that it systematically discusses the application of TENG-based self-powered sensors for environmental monitoring. TENG-based self-powered sensors are classified into two types: TENG as a power supply for professional biochemical sensors and active self-powered sensors where TENG acts as both power source and sensing unit. This paper illustrates the applications of these devices in detecting targets in the environment, such as heavy metal ions, toxic gases, bacterial DNA, and bacteria, and summarizes the relevant performance parameters. It also analyzes key challenges including efficient mechanical energy harvesting, material durability and sensing specificity. Finally, the outlook notes that TENG-based sensors will expand detection ranges and integrate with other technologies, providing valuable guidance for their environmental monitoring applications.

## 1. Introduction

As people’s attention to the living environment continues to rise, various types of sensors have been widely applied in pollutant monitoring. However, the surge in the number of sensors has also brought about an urgent power supply challenge that needs to be addressed. On one hand, supplying power to sensors in remote environments through the power grid incurs high costs; on the other hand, relying on devices such as batteries for power not only necessitates periodic recharging but also raises environmental disposal issues associated with spent batteries. Therefore, if renewable energy sources are widely available in the environment, such as mechanical energy and solar energy, they can be harnessed to power sensors; developing self-powered sensor technology will hold significant practical importance [1,2,3,4,5,6,7]. Triboelectric nanogenerators (TENGs), which can collect the mechanical energy in the environment and convert it into electrical energy to power the sensors, provide a new idea for the development of self-powered sensors for environmental monitoring [8,9,10,11,12,13,14,15]. Its core feature is that it can work efficiently under low-frequency conditions, with advantages such as low cost, simple and lightweight structure, a wide range of material options, and ease of integration [16,17,18,19,20,21,22,23,24,25,26,27,28,29,30,31,32].

Given that the majority of the self-powered environmental sensors are based on the TENG principle, especially the active self-powered sensors, this paper systematically summarizes self-powered environmental sensors based on TENGs in recent years. Firstly, it briefly introduces the fundamental working principles of representative TENGs. Subsequently, based on the different roles that TENGs play within the systems, this paper further categorizes these sensors. They are primarily divided into two major types: one type involves harvesting mechanical energy from the environment using TENGs, converting it into electrical energy, and then supplying it to specialized biochemical sensors for detection. The other type utilizes TENGs not only as an energy source but also as a sensor, achieving detection by leveraging changes in the output electrical signals caused by alterations in the charge transfer capability of the triboelectric materials upon interaction with the analyte—namely, active self-powered sensors. Following that, the existing challenges in this field are summarized and prospects are outlined. This review aims to offer valuable guidance to relevant researchers and facilitate the widespread application of self-powered environmental sensors.

## 2. Mechanism

**Figure 1:**
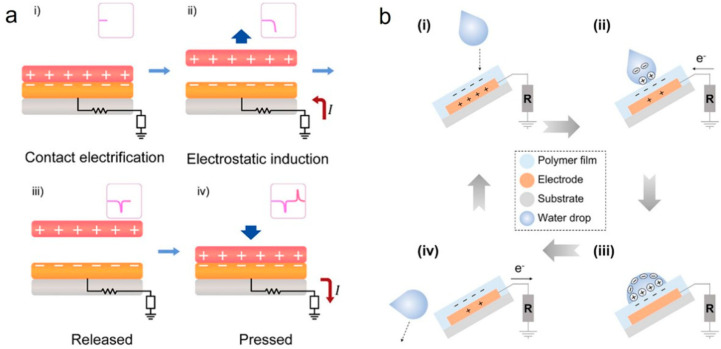
(**a**) Operating principle of a typical TENG with vertical contact-separation mode based on solid–solid interface [45]; copyright 2026, Wiley-VCH. (**b**) Operating principle of a single-electrode mode TENG based on solid–liquid interface [46]. Copyright 2023, Wiley-VCH.

Nowadays, there have been numerous reviews on the working principles of TENGs [33,34,35,36,37,38,39,40,41,42,43,44]. Therefore, in this paper, we simply introduce the working principles by taking two common types of TENGs as examples, which are based on solid–solid interfaces and solid–liquid interfaces, respectively. Firstly, as shown in Figure 1a, the fundamental working mechanism of a solid–solid interface-based TENG is discussed based on two coupled effects: contact electrification and electrostatic induction. When the contact material and the dielectric layer are squeezed and then released by external mechanical forces, contact electrification is triggered. Due to the difference in their electron affinities, positive and negative charged surfaces are generated (Figure 1a(i)). When the two materials separate, the charged surfaces create a potential difference, which induces the flow of electrons through the external circuit (Figure 1a(ii)). When the contact material and the dielectric layer are completely separated, the system reaches an electrostatic equilibrium state (Figure 1a(iii)). When the contact material is squeezed again, the induced electrons flow back from the ground to the electrode (Figure 1a(iv)), generating alternating current under the action of periodic external forces [45].

Figure 1b illustrates the working mechanism of a typical single-electrode liquid–solid TENG. When a water droplet comes into contact with a polymer film, the ionization of surface groups induces negative charges on the polymer, while the water droplet becomes positively charged to maintain electrical neutrality. Due to the dielectric properties and charge retention capability of the polymer film, it can retain the triboelectrically generated negative charges for a longer duration (Figure 1b(i)). Once the water droplet contacts the negatively charged polymer film, the negative charges attract counterions from the water droplet, forming an electric double layer and thereby establishing a positive electric potential difference. Consequently, electrons flow through the external circuit (Figure 1b(ii)) until an equilibrium state is reached (Figure 1b(iii)). This process generates an instantaneous positive current. When the water droplet leaves the polymer surface, a negative electric potential difference is created between the electrode and the ground, causing electrons to transfer from the electrode to the ground until a new equilibrium is established (Figure 1b(iv)) [46].

**Figure 2:**
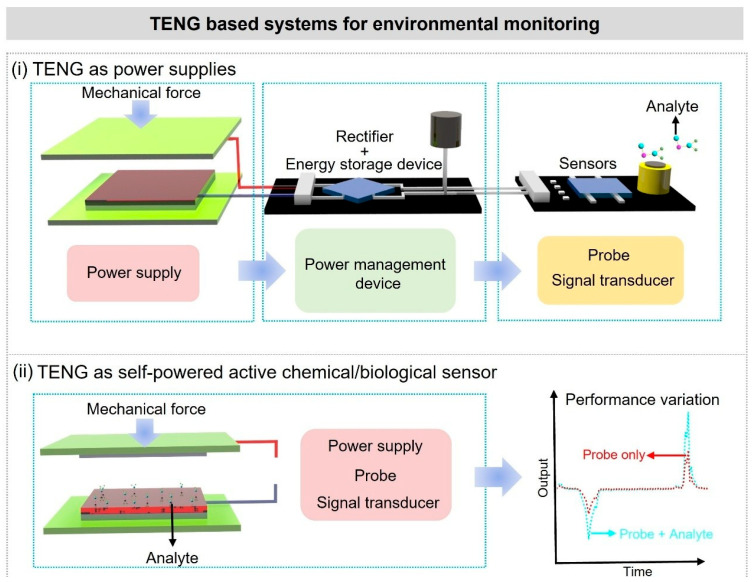
Schematic illustrations of two types of TENG-based self-powered environmental monitoring systems [47]. Copyright 2021, Wiley-VCH.

The schematic diagrams of the two types of self-powered environmental sensors discussed in this paper are shown in Figure 2 [47]. If TENG just works as a power supply for biochemical sensors (Figure 2i), the sensitivity and specificity of the detection are still determined by the professional biochemical sensor itself (Table 1). TENG is only used to ensure the effective supply of electrical energy and its working mechanism is just the same as the traditional TENGs. The critical influence factor for the TENGs’ output performance is the surface potential of the friction material. Since the electricity generated by a TENG is typically an irregular alternating current, powering sensors with it requires the use of a power management unit for rectification and storage [48,49,50,51,52,53,54].

As a contrast, if TENG works as an active self-powered sensor, it serves as both the power source and the sensing unit. For solid–solid interface-based TENG, the identification and sensing of chemical substances on the triboelectric layers can also be explained by the theory of contact electrification and electrostatic induction [55]. Since certain sites on the triboelectric layers of the TENG may be reactive to specific molecules, they are more likely to react with or adsorb these molecules on their surfaces; we refer to these molecules as “target molecules.” In addition to the triboelectric layers themselves, because the electrons of the adsorbed target molecules have different wave functions from those of the atoms on the triboelectric layer, the superposition process varies, thereby affecting the extent of electron cloud overlap. Changes in the extent of overlap influence the number of electrons on each triboelectric layer, thus altering the charge distribution on the triboelectric layers. Furthermore, due to electron accumulation, the electrical potential on the surface of the triboelectric material decreases. The more molecules that are adsorbed, the greater the impact on reducing the energy barrier and electrical potential. The potential changes on both triboelectric layers are reflected in certain electrical characteristics, such as output voltage, output current, or internal resistance. Ultimately, some of these electrical characteristics are utilized to determine the properties of the chemical substances, thereby achieving chemical sensing [56,57]. For a liquid–solid interface-based TENG, the target molecules would also undergo specific reaction or adsorption on the surface of the polymer to alter the overall electrical signal to achieve sensing. However, whether for solid–solid interface-based TENGs or solid–liquid interface-based TENGs, the intrinsic sensing mechanism relies on the specific adsorption and reaction of the friction material to the target. Therefore, the types of targets that can be detected are limited, and the sensitivity and specificity cannot be effectively improved. Taking these considerations into account, currently, researchers try alternative methods, such as ion-selective membranes, enzymes, and probe-assisted sensing mechanisms, to enhance the specificity of the detection.

## 3. Recent Progresses

### 3.1. Self-Powered Environmental Sensing System with TENG as the Power Supply Unit

The approach of harvesting scattered mechanical energy from the environment using TENGs, converting it into electrical energy to power sensors, and thereby enabling the detection of various biochemical substances in the environment holds significant appeal. On one hand, the development of various environmental sensors has reached a relatively mature stage, and TENGs can serve as a robust complement to intermittent renewable energy sources like solar cells, providing stable power to sensors and thus facilitating more reliable self-powered sensing. This section primarily focuses on detection targets at different scales, introducing the recent work on environmental monitoring achieved through the integration of TENGs and sensors.

Heavy metal ions, as a significant type of pollutant, have drawn widespread attention. Among them, heavy metal pollution in water bodies mainly originates from industrial wastewater, mining activities, and so on. It is difficult to naturally degrade, tends to bioaccumulate along the food chain, and poses threats to both the ecosystem and human health [58]. The challenge in achieving self-powered detection of heavy metal ions in water bodies through power supplied by a TENG lies in the fabrication of TENGs capable of harnessing low-frequency wave energy from water bodies. Meanwhile, the sealing performance of TENGs must also be considered to prevent the attenuation of their electrical output performance caused by high humidity. Sharma and his collaborators proposed a self-powered water quality monitoring system based on the integration of a rotary triboelectric nanogenerator (R-TENG) and an AlGaN/GaN high electron mobility transistor (HEMT). This system utilizes polytetrafluoroethylene (PTFE) and nylon as triboelectric layer materials, with aluminum electrodes for electricity collection, and achieves stable underwater operation through magnetic coupling and acrylic encapsulation. The R-TENG delivers an output voltage of 75 V and a current of 7 μA at a rotation speed of 100 rpm, with a maximum output power of 76.64 μW and a power density of 4.97 × 10^−2^ μW/mm^2^. The HEMT sensor, based on the two-dimensional electron gas modulation mechanism, can simultaneously detect pH value (sensitivity of −13.93 mA/pH), heavy metal ions (Pb^2+^, As^3+^, Ca^2+^, with detection limits reaching the nanomolar level), microplastics (polystyrene, 0.001–10 mg/L), and pesticides (chlorpyrifos). This system features complete self-powering, multi-parameter integration, high sensitivity, and strong environmental adaptability, providing an innovative solution for real-time continuous monitoring of complex aquatic environments [59].

In addition to water pollution, air pollution has also drawn widespread attention. Nitrogen dioxide (NO_2_), as a primary gaseous pollutant in the atmosphere, is emitted in large quantities and from diverse sources. It not only causes soil and water pollution and triggers complex atmospheric pollution involving PM_2.5_ and ozone but also strongly irritates the human eyes and respiratory system, potentially leading to fatalities. Moreover, the existing power supply modes for detection present numerous drawbacks. Therefore, precise and rapid detection of nitrogen dioxide is a critical requirement for safeguarding the ecological environment and human health, advancing ecological civilization construction, and addressing atmospheric pollution [60]. Zhang et al. developed a self-powered NO_2_ gas sensor system based on an environmentally friendly triboelectric nanogenerator utilizing leaf texture. This system employs biodegradable gelatin (featuring a leaf-vein-like structure) and PLA/PBAT as triboelectric layers, combined with In_2_O_3_/PPy heterojunction sensing materials, achieving highly sensitive and selective detection of NO_2_ within the range of 5–30 ppm. Through a biomimetic leaf-vein-inspired structural design, the output voltage and current of the TENG were enhanced to 308.7 V and 41.8 μA, respectively, with a peak power density reaching 1386 mW·m^−2^. After rectification and voltage regulation, it outputs a stable 24 V direct current voltage to continuously power the gas sensor. This system demonstrates excellent performance across a wide range of temperatures and humidity levels, featuring rapid response/recovery capabilities and long-term stability, thereby providing a green, self-powered, and efficient integrated solution for environmental pollutant monitoring [61].

**Figure 3:**
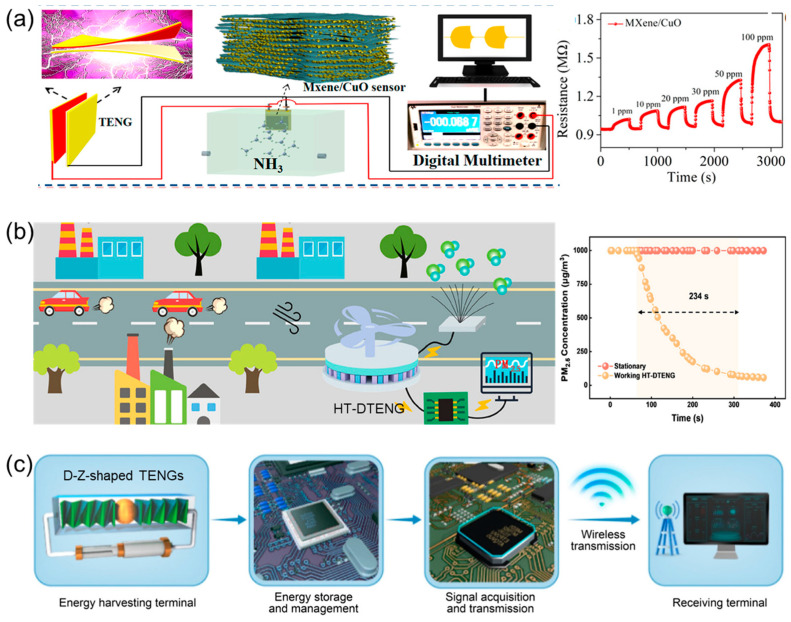
Self-powered systems for detecting (**a**) toxic gases [62]; copyright 2021, American Chemical Society. (**b**) PM_2.5_ [2]; copyright 2025, Wiley-VCH. (**c**) Total dissolved solids [14]; copyright 2024, Wiley-VCH. Powered by TENG.

In addition, ammonia, a toxic gas widely used in agriculture, medicine, and other fields, can cause severe harm to the human body upon leakage, including burns to the skin, eyes, and respiratory mucosa, and even death. Therefore, achieving rapid and precise detection of ammonia is of critical importance and many researchers have devoted themselves to research in this field. For example, Yu and his group report a high-performance TENG-based self-powered ammonia gas sensor (Figure 3a). The sensor employs a three-dimensional layered composite sensing material composed of porous structure MXene and metal–organic framework-derived CuO nanoparticles, which significantly enhances the adsorption and response performance of ammonia gas. The TENG can output a peak-to-peak voltage of 810 V at a working frequency of 10 Hz, with a maximum peak power density of 10.84 W·m^−2^. The integrated self-powered sensor exhibits excellent linear response within the NH_3_ concentration range of 0–100 ppm and can be used for real-time monitoring of NH_3_ release during pork spoilage, demonstrating its potential application in environmental pollutant detection and food safety [62]. Meanwhile, the development of society has also imposed new requirements on ammonia sensors, such as environmental friendliness, wireless connectivity, portability, and self-powering capabilities [63,64]. Zhang and their collaborators reported a biodegradable and environmentally friendly gelatin-based triboelectric nanogenerator for powering a self-powered ammonia (NH_3_) sensor. The innovation of this system lies in the use of a nanostructured gelatin film, prepared via a sandpaper templating method, as the positive triboelectric material, which forms an eco-friendly triboelectric pair with polyimide (PI), offering both high performance and biodegradability. Additionally, a complete self-powered system was constructed, integrating a rectification and voltage regulation circuit with a sensor utilizing PANI/NiCo_2_O_4_ composite NH_3_-sensing material. This system can continuously monitor NH_3_ as a backup power source when the main power supply fails. The gelatin-polyimide-based triboelectric nanogenerator can output a peak voltage of 400 V and a maximum power of 6.16 mW (with a power density of approximately 0.25 mW cm^−2^) under 3 Hz vibration, and after circuit processing, it provides a stable 24 V DC voltage to drive the sensor. The sensor exhibits a good linear response to NH_3_ within the range of 500 ppb–20 ppm (R^2^ = 0.9767), with a detection limit as low as 216 ppb, and demonstrates rapid response/recovery (25 s/59 s @10 ppm), high selectivity, and long-term stability. This work provides a novel strategy for the development of green, self-sustaining environmental gas monitoring systems [65].

As in-depth research is conducted on TENG-based self-powered gas and water detection systems, their application scenarios continue to expand. Chen and his colleagues developed an integrated self-powered environmental purification and monitoring system (Figure 3b). This system innovatively adopts an alternating polarity structure at the friction interface, increasing the output charge density to ±8.18/±8.02 mC·m^−2^ and the energy density to 24.97 J·m^−2^. It also utilizes its negative high-voltage output to drive the carbon fiber array to release high-concentration negative air ions (2.60 × 10^6^ ions·cm^−3^), achieving efficient purification of PM2.5. Moreover, this system can also drive the sensing unit for real-time air quality monitoring [2]. Liu et al. developed a triboelectric nanogenerator-based self-powered sensing platform for environmental and safety monitoring in intelligent mines. To adapt to the complex scenarios in mines, this platform innovatively integrates two functionalized TENG modules: one is a non-contact TENG featuring an arc-edged turbine and a PTFE film, serving as a wind speed sensor. The other is a hybrid-mode TENG based on an FEP film and the principle of vortex-induced vibration, functioning as an energy harvester [66]. Wang et al. built a TENG with a double-helix Z-shaped origami structure, which can achieve continuous sensing of water quality parameters such as total dissolved solids and pH value, as well as wireless signal transmission in marine environments (Figure 3c). The TENG uses FEP and nylon as the negative electrode layer and positive electrode layer of the friction materials, respectively. Based on the double-helix Z-shaped origami structure, low-frequency wave vibration can be efficiently converted into high-frequency mechanical motion, significantly improving the energy collection efficiency. At a wave frequency of 0.8 Hz, this TENG can achieve a peak power density of 55.4 W·m^−3^, an output voltage of 387.3 V, and an output current of 375.2 μA. After integrating an energy management circuit, the TENG can successfully power a commercial water quality detector and an independently designed Zigbee wireless water quality detection module, with a transmission distance of approximately 100 m [14].

In addition to the aforementioned targets, bacteria, which are of larger dimensions, also serve as core indicators in environmental monitoring. They enable rapid early warning of biological contamination risks in water bodies, soil, air, and other environments, providing crucial evidence for assessing environmental health, preventing and controlling public health incidents, and formulating remediation strategies. Qu et al. developed a self-powered biosensing system based on a TENG for the highly sensitive and highly specific detection of bacterial DNA in the environment (targeting the 16S rDNA sequence of sulfate-reducing bacteria (SRB)). This system employs FEP film and aluminum foil as the triboelectric layers, generating a stable output voltage of 160 V. During detection, a “capture probe-target DNA-carbon nanotube signal probe” sandwich structure is formed through DNA hybridization, and signal amplification is achieved by leveraging the high electrical conductivity of carbon nanotubes. The system exhibits a good linear response within the range of 1 pM to 10^5^ pM, with a detection limit as low as 0.084 pM. Finally, by integrating portable modules and LED indicators through 3D printing, it enables self-powered, rapid, and visual on-site DNA detection, providing an innovative passive sensing solution for molecular-level monitoring of environmental pollutants [67]. Other than this work, there are many other related self-powered bacteria sensors that have been published in recent years [68,69,70,71,72,73]. For instance, Wang and their collaborators developed a self-powered biosensing system driven by a TENG for the specific detection of Gram-positive bacteria in the environment (using *Staphylococcus aureus* as an example). This system employs FEP film and aluminum foil as the triboelectric layers, with a stable output voltage of approximately 165 V. It utilizes vancomycin-modified ITO glass (ITO-Van) to specifically capture bacteria and employs guanidino group-functionalized multi-walled carbon nanotubes as a signal amplification material. This enables linear detection of bacterial concentrations within the range of 2 × 10^3^ to 2 × 10^7^ colony-forming units per milliliter (CFU/mL), with a detection limit as low as 2 × 10^3^ CFU/mL, and shows no significant response to Gram-negative bacteria. The system integrates a portable TENG with a LabVIEW-based early warning program, achieving self-powered, real-time detection and visual alarming. It offers an innovative solution for on-site, rapid, and low-power consumption monitoring of environmental pollutants [74].

In addition to the aforementioned representative examples, Table 2 lists the environment monitoring systems powered by TENGs that have been reported in recent years. Based on existing research, such self-powered sensor systems currently still face several challenges.

To meet the complexity of outdoor environments, ensuring the long-term stability of the system is one of the key priorities. From the perspective of triboelectric power generation, long-term stability primarily involves considering the wear and tear of tribomaterials during prolonged operation. To address system stability, innovative approaches in terms of triboelectric material selection and device structure design represent viable solutions. For example, Wang and their collaborators designed a low-wear, multilayered swing-structured TENG composed of a polytetrafluoroethylene (PTFE) film and a rabbit hair brush, aiming to improve space utilization efficiency and thereby enhance output performance. This setup maintained stable output even after 240,000 cycles, demonstrating exceptional durability. The device not only efficiently harvests low-frequency wave energy but also directly powers a water quality detector for self-powered ballast water quality monitoring. Additionally, it achieves electro-dehydration treatment of water-in-oil emulsions by generating a high-voltage electric field (up to approximately 1400 V), with a dehydration rate as high as 99.6%. This work provides an integrated, self-driven energy and sensing solution for the monitoring and management of marine environmental pollutants [79].

In the aforementioned studies, particularly those employing self-developed biochemical sensors rather than commercial ones, there has been relatively little discussion on the specificity of the sensors themselves. However, commercial sensors typically consume more power, and to achieve simultaneous detection of multiple substances, sensor arrays need to be constructed, which places higher demands on the power generation performance of TENGs. Moreover, the detection targets are still limited to standard solutions prepared in laboratories. Therefore, one of the key research focuses for future work in this area is how to achieve automatic sampling and detection in real-world scenarios.

### 3.2. Using TENG as an Active Environmental Sensor for Direct Detection of Targets

In active self-powered sensors, the TENG serves as both an energy supply unit and a sensing unit. The triboelectrification process occurring on the surface of the tribomaterials is the very process that generates the sensing signals. In other words, the target molecules being detected directly participate in the electron gain and loss process during the interaction with the triboelectric materials. Therefore, ensuring the specificity of such devices hinges on the development of tailored triboelectric materials. The performances of the existing active self-powered pollutant detection systems based on intrinsic TENG sensing mechanisms are shown in Table 3 [64,80,81,82,83,84,85,86,87,88,89,90,91,92,93,94,95,96,97,98,99,100,101,102,103].

**Figure 4:**
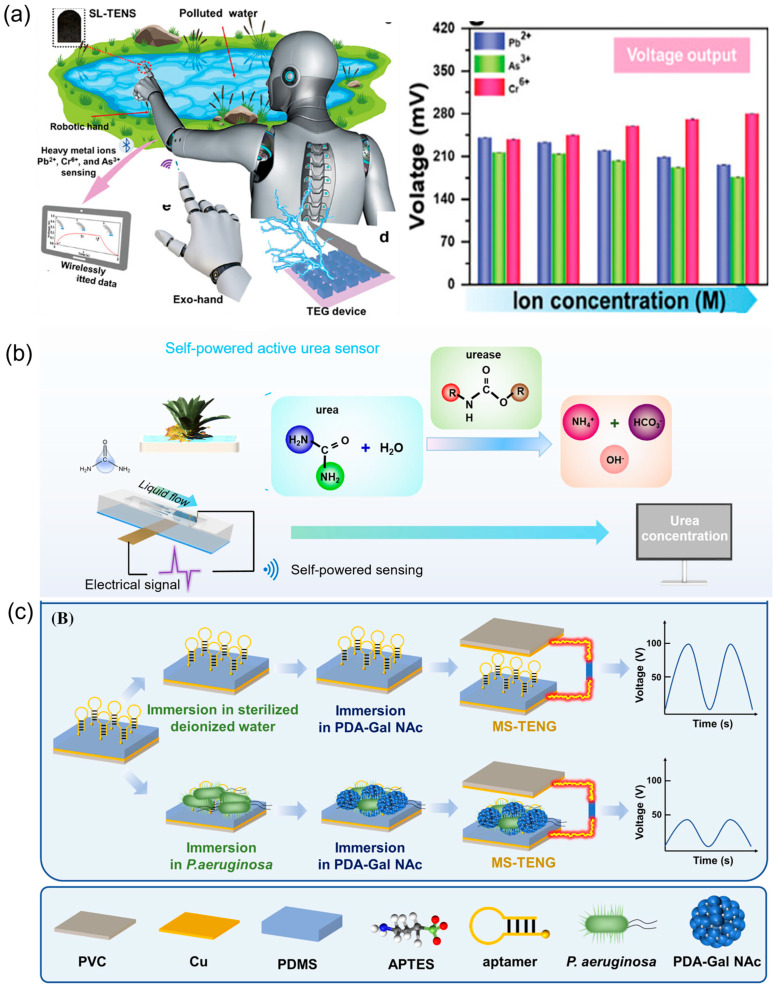
TENG-based active sensors for (**a**) heavy metal ions [80]; copyright 2024, Wiley. (**b**) urea [84]; copyright 2024, Wiley-VCH. (**c**) Bacteria [82]. Copyright 2024, Wiley-VCH.

Huang et al. developed an active, self-powered heavy metal ion detection system centered around a solid–liquid triboelectric sensor (Figure 4a). This system utilizes an array of copper oxide nanowires as the triboelectric material and a copper substrate as the electrode, with ion-selective membranes targeting Pb^2+^, Cr^6+^, and As^3+^ coated on the surface as the sensing material, enabling highly selective detection. The CuO nanowires are chosen based on their cost-effectiveness, abundance, easy synthesis process, chemical stability in aqueous environments, and excellent triboelectric properties. Furthermore, the CuO nanowire-based electrode exhibits highly selective binding affinity with the ions, which can enhance the triboelectric output. The sensor demonstrates a linear response within the concentration range of 10^−11^ to 10^−5^ M, achieving a detection limit at the nanomolar level (5–10 nM). The output voltage varies regularly with ion concentration. This work significantly enhances detection sensitivity and specificity by employing a high-surface-area nanowire structure to boost charge transfer, utilizing ion-selective membranes for specific binding, and directly modulating electrical signals through changes in surface potential and work function induced by ion-membrane binding. By using a printed circuit board with a built-in Bluetooth low energy function, the obtained data can be transmitted wirelessly to a laptop or tablet device. The entire system is autonomously powered by a thermoelectric generator utilizing ambient temperature differentials and wirelessly controlled by an exoskeleton glove to maneuver a robotic arm for safe remote “touch-and-detect” operations. The robotic hand-based sensors exhibit a similar output trend with the corresponding wire sensor. This work provides an efficient and autonomous solution for real-time pollutant monitoring in hazardous environments [80].

In addition to enhancing sensing specificity through the modification of ion-selective materials, for target molecules capable of participating in catalytic reactions, one can leverage the inherent specificity of the catalytic reactions themselves, as well as the dynamic impact of the changing concentrations of reactants and products during the reaction process on the charged state of the friction material’s surface, to achieve specific modulation of the intensity of the output electrical signal. Zhou et al. reported a portable, self-powered microfluidic sensing system based on a triboelectric nanogenerator for the in situ detection of environmental pollutants. This system generates electrical signals through the liquid–solid contact electrification effect between the target liquid in the microfluidic chip and a polydimethylsiloxane (PDMS) triboelectric layer, eliminating the need for an external power supply. The detection process leverages the dynamic impact of the reaction process—where 4-nitrophenol (4-NP) is reduced to 4-aminophenol (4-AP) under the catalysis of gold nanoparticles—on the charged state of the PDMS surface, enabling the detection of 4-nitrophenol [81].

Furthermore, Gao et al. utilized urease, which offers higher specificity, to catalyze the decomposition of urea, thereby achieving specific regulation of the surface charge density of triboelectric materials and enabling the device’s output electrical signal to specifically respond to urea concentration. Meanwhile, to overcome the relatively low electrical signal output of traditional single-electrode mode solid–liquid interface triboelectric generators, they introduced a device structure with a field-effect-transistor-like configuration [101,102], achieving an enhancement in the output electrical signal. Based on this, they developed a self-powered active urea sensor utilizing a triboelectric nanogenerator (Figure 4b). This sensor has been successfully applied to the real-time monitoring of urea concentration changes in nutrient solutions during plant growth, with a detection limit as low as 4 μM and without significant interference from common fertilizers (such as potassium chloride and ammonium dihydrogen phosphate). This technology provides an innovative solution for precision fertilization and resource optimization in smart agriculture, driving the development of the sustainable agricultural Internet of Things [103].

The aforementioned work primarily focuses on enhancing the sensitivity and specificity of solid–liquid interface TENGs. In contrast, active self-powered sensors based on solid–solid interface TENGs generally ensure adequate output electrical signal strength, yet they still lack sufficient specificity. The most effective approach to achieving specificity in such sensors remains the modification with specific probes. Lu et al. developed a bionic dual-recognition strategy for the highly specific detection of Pseudomonas aeruginosa, a pathogenic bacterium in the environment (Figure 4c). First, the bacteria were captured by specific aptamers immobilized on the surface of the PDMS triboelectric layer (primary recognition), followed by the introduction of polydopamine-N-acetyl-D-galactosamine nanoparticles (PDA-GalNAc NPs, secondary recognition) for bacterial binding. Among them, the PDA component significantly reduced the output voltage of the TENG through its electronic properties, enabling signal transduction and amplification. In the absence of bacteria, the output voltage of the sensor was stably maintained at approximately 100 V; it exhibited a linear detection range from 8.7 × 10^3^ to 8.7 × 10^7^ CFU/mL with a low limit of detection (LOD) of 8.7 × 10^3^ CFU/mL. The long-term stability of the TENG is also evaluated by testing the output voltage once a day for 5 consecutive days, which suggests sustained and robust output voltage. The device’s peak-to-peak output voltage consistently stays around 100 V over the 5 days. In addition, the external temperature is also confirmed to have no significant impact on the detection performance of the device. Moreover, no cross-reactivity was observed with *Staphylococcus aureus* and *Escherichia coli*, demonstrating excellent specificity of the sensor. These results reinforce the potential of the device as a reliable microbial detection tool [82].

Based on the aforementioned work, it can be observed that in active self-powered sensing systems, designing sensing mechanisms tailored to specific targets, particularly those with high specificity, constitutes a key research focus and challenge. To address this challenge, the integration of well-established specific probe modification techniques from analytical chemistry with tribomaterials holds promise as a universal approach to enhance specificity. Among these, nucleic acid probes, owing to their inherent versatility, are likely to play a more significant role in future research endeavors [104].

## 4. Conclusions and Outlook

**Figure 5:**
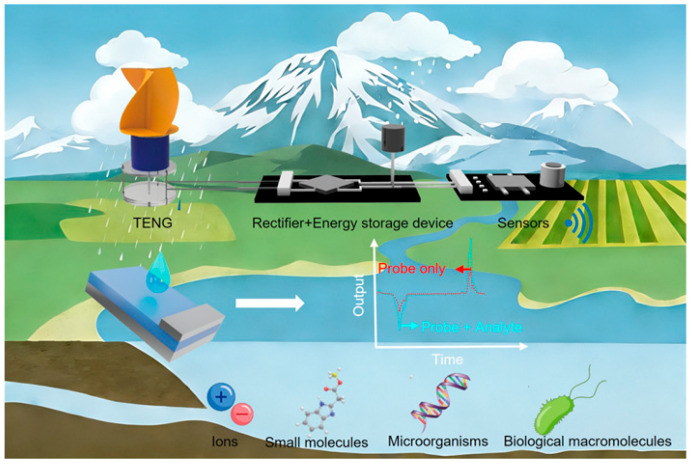
Self-powered environmental monitoring system based on TENGs.

Based on the foregoing review, it is evident that extensive research has been conducted on TENG-based environmental sensors. The two categories of self-powered sensors discussed herein each possess distinct advantages (Figure 5). Firstly, self-powered sensing systems utilizing TENGs as the power supply unit can directly leverage currently mature environmental sensors. The key challenge to be addressed is the efficient harvesting of various forms of irregular mechanical energy by TENGs in complex environments. Furthermore, once the power generation performance and stability of TENGs are improved, the generated electrical energy can not only fulfill detection functions but also enable the direct treatment of pollutants using the surplus energy. For instance, a recent report has demonstrated the use of TENGs as an energy source to achieve both self-powered detection and remediation of heavy metal ions [105].

The advantage of directly employing TENGs as active sensors lies in their relatively simple structure and associated cost benefits. Furthermore, such sensors hold promise for integration with physical sensors, such as pressure sensors, enabling applications in fields like artificial intelligence and robotic tactile sensing. However, the specificity of these sensors primarily relies on the functionalization of the triboelectric material surface. Consequently, tailored development is required for different types of detection targets, which significantly limits their scope of application. Additionally, when these sensors are deployed in outdoor environments, ensuring the uniform distribution of analytes on the triboelectric material surface while simultaneously achieving regular device operation under irregular mechanical energy input presents a further challenge to be addressed.

However, the existing related devices still have certain limitations. For instance, current studies are mainly conducted in controlled laboratory conditions. For real scenarios, the requirements for a device’s environmental interference resistance, sealing property, structural stability and long-term operation are even higher. Thus, practical application performance of the devices still needs to be systematically analyzed and optimized. In addition, at present, there is no standardized evaluation method for the related devices, which makes it difficult to accurately compare and improve their performance, thereby restricting the development of this field. Therefore, standardized evaluation methods should be established. In addition, due to the limitations of the detection principle, the detection target range of the current related devices is still quite limited.

Although there are still many limitations of TENG-based self-powered sensors for environmental monitoring, with the continuous advancement of technology, the self-powered sensors based on TENGs are expected to go further on the path of application and achieve the detection of more targets. For instance, the cooperation with carbon-based materials (e.g., graphite, graphene, and related nanocomposites) may help to ensure efficient charge transfer and surface interactions in self-powered sensing systems [106]. Additionally, controlled sampling may be realized with the integration of microfluidics and signal interpretation can be achieved by coupling with machine learning. Moreover, recent research has already demonstrated the direct application of TENGs for the detection of microplastics [59,106]. As related technologies mature, it is anticipated that a wider range of emerging pollutants will also be amenable to practical self-powered detection.

## Figures and Tables

**Table 1:** Comparison of two kinds of TENG-based self-powered sensors.

Comparison Items	TENG as a Power Sensor	TENG as an Active Sensor
**Sensing Unit**	Professional sensor	TENG
**Sensing Mechanism**	Determined by the professional sensor	Mainly relies on the specific adsorption and reaction of the friction material to the target
**Energy Requirements**	The system has power management unit, so it doesn’t have high requirements for the input energy	The system hasn’t power management unit, so the input energy needs to be stable and effective
**System Complexity**	High complexity: The system needs to incorporate a sensing unit, a TENG power supply unit, and a power management unit	Low complexity: TENG acts as both power source and sensing unit
**Practical Applicability**	The system exhibits high sensitivity and specificity, but with a complex structure. Thus, it is more suitable for application scenarios with higher requirement for detection performance	The system usually exhibits relatively low sensitivity and specificity, but with a simple structure. Thus, it more suitable for application scenarios that have higher requirements for the miniaturization and portability for the devices

**Table 2:** TENG-based environmental energy harvesting systems for powering external circuit.

Triboelectric Material	Electrode Material	Output Voltage (V)	Test Substance	Linear Range	Sensitivity (LOD)	Ref.
FEP, Al	ITO, Al	141.8	pH	2~7	-	[75]
Gelatin, PLA/PBAT	Cu	308.7	NO_2_	5~30 ppm	-	[61]
PVA/Ag nanofibers, FEP	Cu	36	NO_2_	0.5~50 ppm	-	[76]
Ecoflex/MoS_2_/MWCNTs, PU	Cu	1072	NO_2_	1~100 ppm	-	[60]
PTFE, nylon	Al	170	NH_3_	1~100 ppm	1 ppm	[63]
Gelatin, PI	Cu	400	NH_3_	500 ppb~20 ppm	216 ppb	[65]
Latex, PTFE	Cu	810	NH_3_	0~100 ppm	-	[62]
PFA, Au/Cu	ENIG plated Cu electrodes, Cu mass	70	CO_2_	500~4000 ppm	<500 ppm	[77]
silicone/MXene@silicone film, PA66	Cu	1160	C_2_H_5_OH	0.3~20 ppm	0.3 ppm	[78]
PTFE, nylon	Al	75	PS	0.001~10 mg/L	-	[59]
FEP, Al	Al	160	SRB 16S rDNA	1 pM~10^5^ pM	0.084 pM	[67]
FEP, Al	Al	165	*S. aureus*	2 × 10^3^~2 × 10^7^ CFU/mL	2 × 10^3^ CFU/mL	[74]

**Table 3:** TENG sensing mechanisms-based active self-powered pollutant detection systems.

Electrode Material/ Triboelectric Material	Test Substance	Sensitivity (LOD)	Linear Range	Ref.
Cu/CuO NWs	Pb^2+^, Cr^6+^, As^3+^	5 nM~10 nM	10^−11^~10^−5^ M	[80]
Cu/PDMS, BSR-La	F^−^	0.089 mg/L	0.1~0.5 mg/L	[83]
Al/AAO nanopores, PTFE nanowires	Pb^2+^, Cr^3+^, Cu^2+^	-	0~200 µM	[84]
Au/PDMS	Hg^2+^	30 nM	0.1~5 μM	[85]
Al, Sn/FEP	Ag^+^, Mg^2+^, Zn^2+^, Pb^2+^, Cu^2+^, Ni^2+^, Co^2+^	0.01 g/L	0.01~0.53 g/L	[86]
Cu/CuO-ISM, DI water	Hg^2+^	10 pM	10 pM~10 μM	[87]
Cu/PTFE-PDMS	pH	pH = 5	1~5	[88]
Cu/MXene NFs, CA NFs	NH_3_	≤1 ppm	1~100 ppm	[64]
Cu/PTFE	CO_2_	1000 ppm	1000 ppm~200,000 ppm	[89]
Cu/PTFE	HCHO	2 ppm	0~500 ppm	[90]
Cu/PDMS	NO_2_	10 ppb	10 ppb~100 ppb	[91]
Pd/Sb_2_O_3_/PdTe_2_/Si heterojunction	NO_2_, PM_2.5_	200 ppb, 100 μg m^−3^	200 ppb~50 ppm, 100~500 μg m^−3^	[92]
Al/PET, PTFE	CO_2_	700 ppm	700 ppm~12,000 ppm	[93]
Cu/TiO_2_ + PFTS, PTFE + SiO_2_	C_2_H_5_OH	-	-	[94]
Cu/FEP	C_2_H_5_OH	5 ppm	5 ppm~100 ppm	[95]
Cu/BaTiO_3_-PDMS	PS, PE, PVC	8.23 × 10^−8^ wt% 1.26 × 10^−7^ wt% 2.47 × 10^−7^ wt%	0~8 × 10^−6^ wt% 2~8 × 10^−6^ wt% 2~1 × 10^−5^ wt%	[96]
Cu/FEP	PE, PP, PVC, PET, PS	0.0068~0.0223 wt%	0.025~0.25 wt%	[97]
Cu/PDMS	NaCl, C_6_H_5_NO_3_	-	2 × 10^−3^ M~0.1 M	[81]
Cu/PTFE	suspended sediment	-	-	[98]
Cu/X-2Gdm, PTFE	*E. coli*, *S. pneumoniae*	10^6^ CFU/mL	4 × 10^5^~ 4 × 10^8^ CFU/mL	[99]
Al/FEP	sulfate-reducing bacteria	3 × 10^3^ CFU/mL	3 × 10^3^~3 × 10^7^ CFU/mL	[100]
Cu/PDMS	*P. aeruginosa*	8.7 × 10^3^ CFU/mL	8.7 × 10^3^~8.7 × 10^7^ CFU/mL	[82]

## Data Availability

No new data were created or analyzed in this study. Data sharing is not applicable to this article.

## Abbreviations

The following abbreviations are used in this manuscript:

Abbreviation	Full Form
4-AP	4-aminophenol
4-NP	4-nitrophenol
HEMT	High Electron Mobility Transistor
LOD	Limit of Detection
PDA-GalNAc NPs	Polydopamine-N-acetyl-D-galactosamine nanoparticles
PPy	Polypyrrole
R-TENG	Rotary Triboelectric Nanogenerator
SRB	Sulfate-Reducing Bacteria
TENG	Triboelectric Nanogenerator
FEP	Fluorinated Ethylene Propylene
ITO	Indium Tin Oxide
PLA	Polylactic Acid
PBAT	Poly(butylene adipate-co-terephthalate)
PVA	Polyvinyl Alcohol
MWCNTs	Multi-Walled Carbon Nanotubes
PU	Polyurethane
PTFE	Polytetrafluoroethylene
PI	Polyimide
PFA	Perfluoroalkoxy Alkane
ENIG	Electroless Nickel Immersion Gold
PA66	Polyamide 66
PS	Polystyrene
*S. aureus*	*Staphylococcus aureus*
NWs	Nanowires
PDMS	Polydimethylsiloxane
BSR-La	Biosorbent Lanthanum-based material
AAO	Anodic Aluminum Oxide
ISM	Ion-Selective Membrane
NFs	Nanofibers
CA NFs	Cellulose Acetate Nanofibers
PET	Polyethylene terephthalate
PFTS	Perfluorooctyltriethoxysilane
PE	Polyethylene
PVC	Polyvinyl chloride
PP	Polypropylene
*E. coli*	*Escherichia coli*
*S. pneumoniae*	*Streptococcus pneumoniae*
*P. aeruginosa*	*Pseudomonas aeruginosa*
ZIF-8	Zeolitic Imidazolate Framework-8
